# Structural insights into signal transduction of the purinergic receptors P2Y_1_R and P2Y_12_R

**DOI:** 10.1093/procel/pwac025

**Published:** 2022-07-15

**Authors:** Beibei Li, Shuo Han, Mu Wang, Yu Yu, Limin Ma, Xiaojing Chu, Qiuxiang Tan, Qiang Zhao, Beili Wu

**Affiliations:** CAS Key Laboratory of Receptor Research, State Key Laboratory of Drug Research, Shanghai Institute of Materia Medica, Chinese Academy of Sciences, Shanghai 201203, China; University of Chinese Academy of Sciences, Beijing 100049, China; CAS Key Laboratory of Receptor Research, State Key Laboratory of Drug Research, Shanghai Institute of Materia Medica, Chinese Academy of Sciences, Shanghai 201203, China; University of Chinese Academy of Sciences, Beijing 100049, China; CAS Key Laboratory of Receptor Research, State Key Laboratory of Drug Research, Shanghai Institute of Materia Medica, Chinese Academy of Sciences, Shanghai 201203, China; School of Life Science and Technology, ShanghaiTech University, Shanghai 201210, China; CAS Key Laboratory of Receptor Research, State Key Laboratory of Drug Research, Shanghai Institute of Materia Medica, Chinese Academy of Sciences, Shanghai 201203, China; University of Chinese Academy of Sciences, Beijing 100049, China; CAS Key Laboratory of Receptor Research, State Key Laboratory of Drug Research, Shanghai Institute of Materia Medica, Chinese Academy of Sciences, Shanghai 201203, China; CAS Key Laboratory of Receptor Research, State Key Laboratory of Drug Research, Shanghai Institute of Materia Medica, Chinese Academy of Sciences, Shanghai 201203, China; CAS Key Laboratory of Receptor Research, State Key Laboratory of Drug Research, Shanghai Institute of Materia Medica, Chinese Academy of Sciences, Shanghai 201203, China; University of Chinese Academy of Sciences, Beijing 100049, China; CAS Key Laboratory of Receptor Research, State Key Laboratory of Drug Research, Shanghai Institute of Materia Medica, Chinese Academy of Sciences, Shanghai 201203, China; University of Chinese Academy of Sciences, Beijing 100049, China; Zhongshan Institute for Drug Discovery, Shanghai Institute of Materia Medica, Chinese Academy of Sciences, Zhongshan 528400, China; CAS Key Laboratory of Receptor Research, State Key Laboratory of Drug Research, Shanghai Institute of Materia Medica, Chinese Academy of Sciences, Shanghai 201203, China; University of Chinese Academy of Sciences, Beijing 100049, China; School of Life Science and Technology, ShanghaiTech University, Shanghai 201210, China; School of Pharmaceutical Science and Technology, Hangzhou Institute for Advanced Study, UCAS, Hangzhou 310024, China

**Dear Editor**,

The purinergic receptors (P2YRs) are involved in a variety of physiological processes, including proliferation, chemotaxis, cancer metastasis, cardiovascular events, neurodegenerative diseases and aging (Weisman et al., 2012). Thus far, eight human P2YRs have been characterized and are classified into two sub-families based on their sequence homology and signal transduction mechanisms, including P2Y_1_R-like receptors that signal preferentially through G_q/11_ proteins and P2Y_12_R-like receptors that activate G_i/o_ proteins (Abbracchio et al., 2006).

P2Y_1_R and P2Y_12_R, which recognize the same endogenous ligands ADP and ATP, play vital roles in platelet aggregation, making them attractive drug targets for the treatment of thrombotic diseases. P2Y_1_R activation initiates shape change and activation of the platelets (Leon et al., 1999), while P2Y_12_R activation further amplifies this process and results in platelet aggregation (Kim and Kunapuli, 2011). Our previous efforts enabled determination of inactive structures of P2Y_1_R and P2Y_12_R in complex with various antagonists (Zhang et al., 2014b, 2015) and structures of P2Y_12_R bound to the agonist 2-methylthio-adenosine-5ʹ-diphosphate (2MeSADP) or 2-methylthio-adenosine-5ʹ-triphosphate (2MeSATP) in absence of G protein (Zhang et al., 2014a). However, lacking a fully active structure, molecular mechanism of P2YR signal transduction remains elusive. Thus, we solved the fully active structures of P2Y_1_R and P2Y_12_R bound to the agonist 2MeSADP and different classes of heterotrimeric G proteins, G_11_ and G_i2_, respectively (the antibody scFv16 was used to stabilize the G_11_ protein) (Figs. 1A, 1B, S1 and S2; Table S1). The new structural details, together with functional data, uncover key factors that govern signal recognition, activation modulation, and G-protein selectivity of the two representative P2YRs.

**Figure 1.**
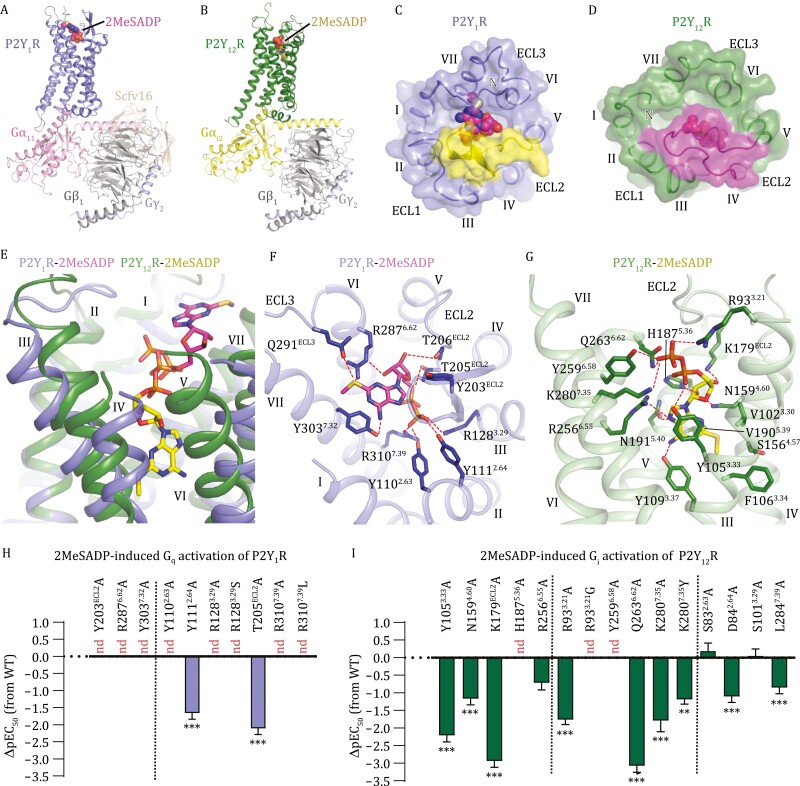
**Overall structures and ligand-binding modes of the 2MeSADP-P2Y**_**1**_ **R-G**_**11**_ **and 2MeSADP-P2Y**_**12**_ **R-G**_**i2**_ **complexes.** (A) Structure of the 2MeSADP-P2Y_1_R-G_11_ complex. The structure is shown in cartoon representation. The agonist 2MeSADP is shown as spheres. (B) Structure of the 2MeSADP-P2Y_12_R-G_i2_ complex. The structure is shown in cartoon representation. The agonist 2MeSADP is shown as spheres. (C) Ligand-binding pocket in the 2MeSADP-P2Y_1_R-G_11_ complex. The receptor is shown in cartoon and surface representations in an extracellular view. ECL2 is colored yellow. 2MeSADP is shown as spheres. (D) Ligand-binding pocket in the 2MeSADP-P2Y_12_R-G_i2_ complex. The receptor is shown in cartoon and surface representations in an extracellular view. ECL2 is colored magenta. 2MeSADP is shown as spheres. (E) Comparison of 2MeSADP binding poses at P2Y_1_R and P2Y_12_R. The agonist 2MeSADP is shown as sticks. The receptors are shown as cartoon. (F) Interactions between P2Y_1_R and 2MeSADP. The P2Y_1_R residues involved in interactions are shown as blue sticks. The polar interactions are displayed as red dashed lines. (G) Interactions between P2Y_12_R and 2MeSADP. The P2Y_12_R residues involved in interactions are shown as green sticks. The polar interactions are displayed as red dashed lines. (H and I) 2MeSADP-induced G-protein activation assays of P2Y_1_R (H) and P2Y_12_R (I). Bars represent differences in calculated 2MeSADP potency (pEC_50_) for each mutant relative to the wild-type receptor (WT). Data are shown as mean ± s.e.m. from at least three independent experiments performed in triplicate. nd, not determined. **P < 0.01, ***P < 0.001 (one-way ANOVA followed by Dunnett’s post-test, compared with the response of WT). See Table S2 for detailed statistical evaluation and expression levels.

The 2MeSADP-P2Y_1_R-G_11_ and 2MeSADP-P2Y_12_R-G_i2_ complexes are structurally similar with C_α_ root-mean-square deviation (RMSD) of 2.3 Å (Fig. S3A and S3B). Despite recognizing different G-protein subtypes, P2Y_1_R and P2Y_12_R exhibit a similar backbone conformation of the helical bundle in the intracellular region (Fig. S3B). Compared to the inactive structures, the outward movement of helix VI that represents a hallmark of receptor activation in class A and B G-protein-coupled receptors (GPCRs) (Rasmussen et al., 2011; Qiao et al., 2020) was observed in the G-protein-bound P2Y_1_R and P2Y_12_R structures (Fig. S3C and S3D). The largest deviation occurs in the extracellular regions of the receptors. The second extracellular loop (ECL2) in P2Y_1_R adopts a β-hairpin structure, with its C-terminal region embedded at the bottom of the binding site for 2MeSADP, while P2Y_12_R’s ECL2 exhibits a relatively disordered conformation and caps the ligand-binding pocket (Fig. 1C and 1D). The different conformations of ECL2 are most likely associated with the distinct binding modes of 2MeSADP at these two receptors.

In P2Y_1_R and P2Y_12_R, the agonist 2MeSADP exhibits different binding poses and the binding sites only partially overlap in the diphosphate region (Fig. 1E). Upon binding to P2Y_1_R, the agonist occupies an upper binding site in the receptor extracellular region. Its adenine group points towards the extracellular surface and makes contacts with helices VI and VII, ECL2, and the third extracellular loop (ECL3) of the receptor (Fig. 1F). In contrast, the adenine group penetrates deep into a narrow binding cavity within the receptor transmembrane core when bound to P2Y_12_R, forming extensive interactions with helices III-VI (Fig. 1G). Different binding modes were also observed for the ribose moiety. In the P2Y_1_R-bound state, the ribose ring packs against ECL2, while it occupies a small cavity shaped by helices III-V and ECL2 in P2Y_12_R (Fig. 1F and 1G). The distinct binding modes are supported by our functional data of a bioluminescence resonance energy transfer (BRET) assay using TRUPATH biosensors (Olsen et al., 2020), in which the mutations Y203A, R287^6.62^A, and Y303^7.32^A (superscripts indicate Ballesteros-Weinstein residue numbering) of P2Y_1_R dramatically impaired 2MeSADP-induced G_q_ activation and the alanine replacements of Y105^3.33^, N159^4.60^A, K179A, H187^5.36^, and R256^6.55^ substantially reduced the agonist potency of 2MeSADP in inducing G_i_ activation of P2Y_12_R (Figs. 1H, 1I and S4A–C; Table S2).

The 5ʹ-phosphate has been proved important for both binding affinity and agonist efficacy of nucleotide ligands at P2Y_1_R and P2Y_12_R (Gao et al., 2008). Intriguingly, this negatively charged group in the agonist binds to the two P2YRs in a receptor-specific manner. At P2Y_1_R, the 5ʹ-diphosphate is anchored in the extracellular region of the receptor by two salt bridges with R128^3.29^ and R310^7.39^ and a hydrogen-bond network formed with Y110^2.63^, Y111^2.64^, and T205 in the extracellular tip of helix II and ECL2 (Fig. 1F). The importance of these interactions in mediating the 2MeSADP-induced receptor activation is reflected by an over 46-fold reduction of the agonist potency for the alanine mutants of these key residues (Figs. 1H and S4D; Table S2). In P2Y_12_R, these residues are substituted by S83^2.63^, D84^2.64^, S101^3.29^, and L284^7.39^, which disrupt the polar interaction network and exclude the possibility that the same site in P2Y_12_R accommodates the diphosphate of 2MeSADP. Alternatively, the 5ʹ-diphosphate group is engaged in two salt bridges with R93^3.21^ and K280^7.35^ and three hydrogen bonds with Y105^3.33^, Y259^6.58^, and Q263^6.62^ in helices III, VI, and VII of P2Y_12_R (Fig. 1G). This binding mode agrees with an over 60-fold reduction of agonist potency (EC_50_) or an 80% decrease of maximal response (*E*_max_) of the 2MeSADP-induced G_i_ activation for the P2Y_12_R mutants R93^3.21^A, Y105^3.33^A, Y259^6.58^A, Q263^6.62^A, and K280^7.35^A but a limited effect of S83^2.63^A, D84^2.64^A, S101^3.29^A, and L284^7.39^A on receptor activation (<13-fold reduction of EC_50_) (Fig. 1I, S4E and S4F; Table S2).

Sequence alignment of the human P2YRs displays a sub-family-conserved manner of the key basic residues of P2Y_1_R and P2Y_12_R that form ionic interactions with the 5ʹ-diphosphate group of 2MeSADP. The P2Y_1_R residues R^3.29^ and R^7.39^ are conserved in all the P2Y_1_R-like receptors but substituted by S/A^3.29^ and L^7.39^, respectively, in the P2Y_12_R-like receptors (Fig. S5). Similarly, the residues at positions 3.21 and 7.35 are positively charged in the P2Y_12_R sub-family (R^3.21^ and K^7.35^, except for N^3.21^ in P2Y_14_R), while in the P2Y_1_R sub-family these residues are non-charged (G/S^3.21^ and Y^7.35^) (Fig. S5). The non-charged substitutions most likely disturb the recognition between the receptor and the functionally important 5ʹ-diphosphate group of the agonist. This is supported by a notable detrimental effect of the P2Y_1_R mutations R128^3.29^S and R310^7.39^L and the P2Y_12_R mutations R93^3.21^G and K280^7.35^Y on the 2MeSADP-induced G-protein activation (Figs. 1H, 1I, S4D and S4E; Table S2). These data suggest that the different charge distribution within the ligand-binding pocket accounts for the distinct binding modes of 2MeSADP at P2Y_1_R and P2Y_12_R, and the receptors within the same P2YR sub-family may adopt a similar binding pattern in recognition of the 5ʹ-phosphate of the nucleotide agonists.

It has been acknowledged that activation of class A GPCRs is associated with conformational rearrangements of some conserved structural elements, including the so-called “toggle switch” W^6.48^ (Koehl et al., 2018). Unlike the other class A GPCRs, most of the receptors in the δ-family including the purinergic receptors have a less bulky residue at the position 6.48 (F/Y/L^6.48^). The alanine and tryptophan replacements of Y/F^6.48^ in P2Y_1_R and P2Y_12_R have little effect on the 2MeSADP-induced G-protein activation (Fig. S4G and S4H; Table S2), demonstrating that this residue is not essential for activation of these two P2YRs. These data suggest that P2Y_1_R and P2Y_12_R may adopt an activation mode different from the other class A receptors.

The 2MeSADP-P2Y_1_R-G_11_ structure and the previously determined inactive P2Y_1_R structure (Zhang et al., 2015) reveal that the agonist 2MeSADP and nucleotide-like antagonist MRS2500 occupy a similar binding pocket but display distinct binding modes (Fig. S3E). The adenine ring of MRS2500 inserts into a binding crevice between the N-terminal region and helix VI of P2Y_1_R. In the active structure the adenine ring of 2MeSADP rotates by 90° and moves towards the receptor N terminus. It packs tightly against helix VII, pushing Y303^7.32^ and Y306^7.35^ to move downwards by about 2 Å and subsequently leading to a 1-Å outward shift of residue F276^6.51^ (Fig. 2A). This likely results in the outward movement of helix VI on the intracellular side, which is required for G-protein coupling. Furthermore, on the opposite side of the ligand-binding pocket, the ionic interaction between the 5ʹ-diphosphate group of 2MeSADP and R128^3.29^, which does not exist in the MRS2500-bound structure, pushes helix III away from the central axis of the helical bundle (Fig. 2A). This movement breaks the hydrophobic interaction core formed by F131^3.32^, L135^3.36^, and F276^6.51^, which destabilizes the inactive conformation of the receptor core and facilitates the conformational rearrangement of helix VI upon activation (Fig. S3C). Consistent with these findings, our functional data showed a substantial loss of G_q_ activation for the P2Y_1_R mutants F131^3.32^A, F276^6.51^A, Y303^7.32^A, and Y306^7.35^A (Fig. S4I and Table S2).

**Figure 2.**
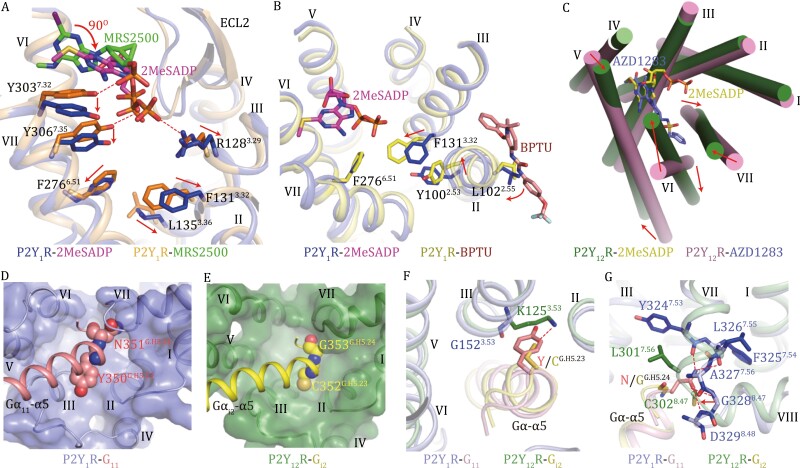
**2MeSADP-induced activation and G-protein-binding pockets of P2Y**_**1**_ **R and P2Y**_**12**_ **R.** (A) Comparison between the active 2MeSADP-P2Y_1_R-G_11_ structure and the inactive P2Y_1_R-MRS2500 structure (PDB ID: 4XNW). The key residues involved in modulating receptor activation are shown as sticks. The red arrows indicate the conformational changes of the key residues in the active structure relative to the inactive structure. The hydrogen bonds between MRS2500 and the P2Y_1_R residues Y303^7.32^ and Y306^7.35^ as well as between 2MeSADP and the P2Y_1_R residue R128^3.29^ are shown as red dashed lines. (B) Comparison between the active 2MeSADP-P2Y_1_R-G_11_ structure and the inactive P2Y_1_R-BPTU structure (PDB ID: 4XNV). The key residues involved in modulating receptor activation are shown as sticks. The red arrows indicate the conformational changes of the key residues in the inactive structure relative to the active structure. (C) Comparison between the active 2MeSADP-P2Y_12_R-G_i2_ structure and the inactive P2Y_12_R-AZD1283 structure (PDB ID: 4NTJ). The receptor helical bundles in the two structures are shown in an extracellular view. The red arrows indicate the movements of helices V, VI, and VII on both extracellular and intracellular sides. (D) Binding pocket for the α5 helix of Gα_11_ in P2Y_1_R. The Gα_11_ residues Y350^G.H5.23^ and N351^G.H5.24^ are shown as spheres. The receptor is shown in cartoon and surface representations in an intracellular view. (E) Binding pocket for the α5 helix of Gα_i2_ in P2Y_12_R. The Gα_i2_ residues C352^G.H5.23^ and G353^G.H5.24^ are shown as spheres. The receptor is shown in cartoon and surface representations in an intracellular view. (F) Interactions between the receptors and the Gα residue at position G.H5.23. The residues involved in interactions are shown as sticks. The hydrogen bond between K125^3.53^ of P2Y_12_R and C^G.H5.23^ of Gα_i2_ α5 is displayed as a red dashed line. (G) Interactions between the receptors and the Gα residue at position G.H5.24. The residues involved in interactions are shown as sticks. The hydrogen bonds between P2Y_1_R and the Gα_11_ residue N351^G.H5.24^ are shown as red dashed lines. The red arrow indicates the movement of the linker region between helices VII and VIII in P2Y_12_R relative to that in P2Y_1_R.

In the MRS2500-bound P2Y_1_R structure, the antagonist forms interactions with the residues Y303^7.32^ and Y306^7.35^, constraining their conformational changes and stabilizing the receptor in the inactive state (Fig. 2A). The non-nucleotide antagonist BPTU occupies an allosteric binding pocket on the external receptor interface with the lipid bilayer, making contacts with helices I, II, and III of the receptor (Zhang et al., 2015). Comparison of the 2MeSADP- and BPTU-bound structures revels that BPTU would form a clash with helix III if the helix were in the same conformation as that observed in the active structure (Fig. 2B). As such, BPTU limits the rearrangement of helix III and stabilizes the inward conformation of F131^3.32^. In addition, the interaction between BPTU and L102^2.55^ induces a clockwise rotation of helix II (~ 30 degrees, extracellular view), which results in a shift (for 1.4 Å) of the side chain of Y100^2.53^ towards helix III, causing an additional spatial hindrance to block the conformational change of F131^3.32^ (Fig. 2B). At the inward position, the residue F131^3.32^ stabilizes the receptor in the inactive state by forming the interaction with F276^6.51^ to constrain the conformational change of helix VI (Fig. 2B).

In contrast to P2Y_1_R, where the receptor activation is regulated mainly through repositioning of residue side chains within the ligand-binding pocket, P2Y_12_R modulates its activity in a more global way. Compared to the antagonist AZD1283-bound inactive structure, the extracellular ends of helices VI and VII display large inward displacements upon binding to 2MeSADP (Fig. 2C). This conformational change in the extracellular binding pocket is most likely associated with the outward movements of helices VI and VII in the intracellular region, and thereby creates a cavity for G-protein accommodation (Fig. 2C). Furthermore, due to interactions between 2MeSADP and helix V, the extracellular part of helix V in P2Y_12_R slightly shifts towards helix VI by 2.6 Å (Fig. 2C). Lacking the conserved residue P^5.50^ (N^5.50^ in P2Y_12_R), this helix exhibits a straight conformation with rigidity that facilitates relaying the conformational change in the extracellular region to the intracellular side. Thus, the conformational change of helix V in the ligand-binding pocket is likely linked with a movement of its intracellular region towards helix III, which enables its interaction with the G_i_ protein (Figs. 2C and S3D). The antagonist AZD1283, which partially shares the binding site with 2MeSADP, forms a spatial hindrance to block the inward movements of helices VI and VII and thus inhibits receptor function (Fig. S3F). In contrast to the small-molecule antagonists of P2Y_1_R and P2Y_12_R, which impair receptor activation by constraining the conformational change of the helical bundle, the previously reported antibody-based inhibitors of these two receptors (Karim et al., 2015; Hensch et al., 2017) most likely block agonist binding/entry by forming a spatial hindrance through interactions with the receptor extracellular loops.

Despite the same endogenous ligand, P2Y_1_R and P2Y_12_R couple to different G-protein subtypes, raising a question about G-protein selectivity. The 2MeSADP-P2Y_1_R-G_11_ and 2MeSADP-P2Y_12_R-G_i2_ structures display a similar conformation of the intracellular half of the receptor helical bundle (Supplementary Fig. S3B), suggesting that receptor backbone architecture unlikely accounts for the G-protein coupling preference. The α5 helix at the C termini of Gα_11_ and Gα_i_, a key region that binds to the receptor intracellular binding cavity, shows sequence diversity at positions G.H5.23 and G.H5.24 [common Gα numbering system (Flock et al., 2015)]. The bulky residues Y^G.H5.23^ and N^G.H5.24^ of Gα_11_ (C^G.H5.23^ and G^G.H5.24^ in Gα_i_) require a larger space in the binding cavity to place their side chains (Fig. 2D and 2E). Indeed, to accommodate the Gα_11_ residue Y350^G.H5.23^, P2Y_1_R has a glycine at position 3.53, while in P2Y_12_R the counterpart is K125^3.53^ that facilitates a polar interaction with C352^G.H5.23^ in Gα_i_ (Fig. 2F). The requirement of spare room is supported by a notable loss of 2MeSADP-induced G_q_ activation for the P2Y_1_R mutant G152^3.53^F (Fig. S4I; Table S2). A similar spatial preference also occurs at the Gα residue G.H5.24, which is associated with different conformations of the linker region between helices VII and VIII in P2Y_1_R and P2Y_12_R. In the 2MeSADP-P2Y_1_R-G_11_ complex, the side chain of N351^G.H5.24^ in Gα_11_ forms multiple hydrogen bonds with the main chain of Y324^7.53^-D329^8.48^ in P2Y_1_R (Fig. 2G). For P2Y_12_R, the residues L301^7.56^ and C302^8.47^ in the linker region move towards the C terminus of the Gα α5 helix, packing tightly against the main chain of G353^G.H5.24^ in Gα_i2_ (Fig. 2G). These differences demonstrate the importance of spatial constrains in determining G-protein specificity. However, this does not rule out the possibility that a global conformational change and/or specific intracellular conformations are required for initial G-protein recognition, which may also account for G-protein specificity.

Taken together, this work provides a detailed molecular map of distinct ligand-binding patterns, non-canonical activation modes and pleiotropic G-protein coupling of the physiologically important purinergic receptors P2Y_1_R and P2Y_12_R, which deepens our understanding about the molecular mechanisms of P2YR functionality and GPCR signal transduction.

## Supplementary Material

pwac025_suppl_Supplementary_Material

## Footnotes

The cryo-EM studies were performed at the electron microscopy facility of Shanghai Institute of Materia Medica (SIMM), Chinese Academy of Sciences. We thank Q. Wang from SIMM for cryo-EM data collection.

This work was supported by the National Science Foundation of China grants (31825010 and 82121005), National Key R&D Program of China (2018YFA0507000), CAS Strategic Priority Research Program (XDB37030100), and Shanghai Pilot Program for Basic Research-Chinese Academy of Sciences, Shanghai Branch (JCYJ-SHFY-2021-008). The authors declare no conflict of interest. This article does not contain any studies with human or animal subjects performed by any of the authors. All authors give their consent for the publication of all materials within the text in Protein & Cell.

Atomic coordinates and cryo-EM density maps for the structures of 2MeSADP-P2Y1R-G11, 2MeSADP-P2Y12R-Gi2 with identification codes 7XXH and 7XXI, and the Electron Microscopy Data Bank (EMDB) under accession codes EMD-33503 and EMD-33504. The authors declare that all other relevant data supporting the findings of this study is available within the paper and its Supplemental materials, or from the corresponding authors upon request. All the experimental materials generated in this study are available from the corresponding authors upon reasonable request.

B.L. developed the protein expression and purification procedures, and prepared the protein samples for cryo-EM. S.H. and M.W. prepared cryo-samples, collected cryo-EM data, performed cryo-EM data processing and analysis, and built the initial models. Y.Y. helped with protein preparation and functional assays. L.M. and X.C. expressed the proteins. Q.T. refined the structures, designed and performed the functional assays, and wrote the first draft. Q.Z. and B.W. initiated the project, planned and analyzed experiments, supervised the research, and wrote the manuscript with input from all co-authors.
