# Supplementary material for: Structural insights into signal transduction of the purinergic receptors P2Y1R and P2Y12R
Source: Protein Cell. 2022 Jul 15;14(5):382–6. doi: 10.1093/procel/pwac025 (PMC10166156; doi:10.1093/procel/pwac025)
Supplement: pwac025_suppl_Supplementary_Material [file pwac025_suppl_supplementary_material.pdf]

Supplementary Materials

Structural insights into signal transduction of the purinergic receptors $P2Y_1R$ and $P2Y_{12}R$

Beibei Li[1,2#], Shuo Han[1,2#], Mu Wang[1,3], Yu Yu[1,2], Limin Ma[1], Xiaojing Chu[1], Qiuxiang Tan[1,2]*, Qiang Zhao[1,2,4]*, Beili Wu[1,2,3,5]*

[1]CAS Key Laboratory of Receptor Research, State Key Laboratory of Drug Research, Shanghai Institute of Materia Medica, Chinese Academy of Sciences, Shanghai 201203, China.

[2]University of Chinese Academy of Sciences, Beijing 100101, China.

[3]School of Life Science and Technology, ShanghaiTech University, Shanghai 201210, China.

[4]Zhongshan Institute for Drug Discovery, Shanghai Institute of Materia Medica, Chinese Academy of Sciences, Zhongshan 528400, China.

[5]School of Pharmaceutical Science and Technology, Hangzhou Institute for Advanced Study, UCAS, Hangzhou 310024, China.

[#]Beibei Li and Shuo Han contributed equally to this work.

*Correspondence: qxtan@simm.ac.cn (Q. Tan), zhaoq@simm.ac.cn (Q. Zhao), beiliwu@simm.ac.cn (B. Wu)

MATERIALS AND METHODS

Construct cloning and expression of $P2Y_1R–G_{11}$ and $P2Y_{12}R–G_{i2}$ complexes

The wild-type human $P2Y_1R$ and $P2Y_{12}R$ genes were cloned into a modified pFastBac1 vector with haemagglutinin (HA) signal peptide and a flag epitope tag (DYKDDDD) at the N terminus and an HRV-3C cleavage site and a 2×strep tag at the C terminus. The genes of the Gα ($G\alpha_{11}$ and $G\alpha_{i2}$) and $G\beta_1\gamma_2$ subunits were cloned into the pFastbac1 and pFastDual vectors, respectively. The first 24 residues at the N terminus of $G\alpha_{11}$ were replaced with the counterpart in G_{i1} to impart the ability of binding to scFv16, the single-chain variable fragment of mAb16, which stabilizes the nucleotide-free GPCR–G protein complex (Maeda et al., 2018). A dominant-negative form of $G\alpha_{i2}$ ($DNG\alpha_{i2}$) was generated by introducing five mutations, S47C, G202T, G203A, E245A and A326S (Draper-Joyce et al., 2018).

The recombinant baculoviruses of the receptors, G proteins and Ric8a were generated using Bac-to-Bac baculovirus expression system (Invitrogen). The $P2Y_1R$ and G_{11} heterotrimer were co-expressed in *Spodoptera frugiperda* (*Sf*9) insect cells (Invitrogen), which were grown in ESF 921™ medium (Expression systems) at 27 ºC. The cells at a density of 2.0×10^6 cells per ml were infected with the virus preparations for $P2Y_1R$, $G\alpha_{11}$, $G\beta_1\gamma_2$ and Ric8a at a multiplicity of infection (MOI) ratio of 1:1:1:1. The $P2Y_{12}R$ and G_{i2} heterotrimer were co-expressed in High Five insect cells (Invitrogen). The cells at a density of 1.5×10^6 cells per ml were infected with the $P2Y_{12}R$, $G\alpha_{i2}$ and $G\beta_1\gamma_2$ viral stocks at an MOI ratio of 1:1:1. The infected cells were cultured at 27 ºC for 48 h before harvested by centrifugation and the cell pellets were stored at –80 ºC for further use.

Construct cloning, expression and purification of scFv16

The scFv16 gene was cloned into a modified pFastBac1 vector containing a gp67 secretion signal peptide at the N terminus and a 6×His tag at the C terminus. ScFv16 was expressed in the Hive Five insect cells using the Bac-to-Bac baculovirus expression system (Invitrogen). The cells at a density of 1.5×10^6 cells per ml were infected with the scFv16 viral stock at an MOI of 5. The infected cells were cultured at 27 ºC for 48 h. The culture supernatant was collected by centrifugation and pH balanced to pH 8.0 by adding 1M Tris-HCl, pH 8.0, 1mM $NiCl_2$ and 5 mM $CaCl_2$. The protein sample was then loaded to a Ni-NTA column (Qiagen) and incubated at 4 ºC for 1 h. The column was washed with 10 column volumes of wash buffer 1 containing 20 mM HEPES, pH 7.5, 500 mM NaCl and 10 mM imidazole followed by 10 column volumes of wash buffer 2 containing 20 mM HEPES, pH 7.5, 100 mM NaCl and 10 mM imidazole. The protein was then eluted with 5 column volumes of wash buffer 2 supplemented with 250 mM imidazole. The C-terminal His tag was cleaved with His-tagged HRV-3C protease (custom-made) at 4 ºC overnight. The protease and cleaved His tag were removed by reverse binding using Ni-NTA resin (Qiagen) for 1 h. The flow through was collected and further purified with gel filtration chromatography using a Superdex 10/300 column (GE Healthcare) pre-equilibrated with 20 mM HEPES, pH 7.5 and 100 mM NaCl. The monomeric fractions were pooled, concentrated and flash-frozen in liquid nitrogen, and then stored at –80 ºC for further use.

Purification of 2MeSADP–P2Y$_1$R–G$_{11}$ and 2MeSADP–P2Y$_{12}$R–G$_{i2}$ complexes

The cells that express the P2Y$_1$R–G$_{11}$ complex were thawed and lysed in a hypotonic buffer containing 20 mM HEPES, pH7.4, 50 mM NaCl, 2 mM $MgCl_2$ with addition of EDTA-free complete protease inhibitor cocktail tablets (Roche). The 2MeSADP–P2Y$_1$R–G$_{11}$ complex was formed in the membranes by adding 2MeSADP to a final concentration of 10 μM in presence of 20 μg ml^{-1} scFv16 and 2 units of apyrase (NEB). The mixture was incubated for 1 h at room

temperature and the membranes were collected by centrifugation at 40,000*g* for 30 min. The membranes were then solubilized in a buffer containing 20 mM HEPES, pH 7.4, 50 mM NaCl, 2 mM $MgCl_2$, 0.5% (w/v) *n*-dodecyl-β-D-maltopyranoside (DDM, Anatrace), 0.1% (w/v) cholesterol hemisuccinate (CHS, Sigma-Aldrich), 10 μM 2MeSADP, 100 μM tris(2-carboxyethyl)phosphine (TCEP) and 2 units of apyrase (NEB) at 4 ºC for 3 h. The supernatant was isolated by ultracentrifugation at 40,000*g* for 30 min and incubated with strep resin (IBA Lifesciences) overnight at 4 ºC. The resin was washed with 10 column volumes of wash buffer 1 containing 20 mM HEPES, pH 7.4, 100 mM NaCl, 2 mM $MgCl_2$, 0.05% DDM, 0.01% CHS, 100 μM TCEP and 30 μM 2MeSADP. The detergent was exchanged by incubating the resin with wash buffer 1 supplemented with 0.25% (w/v) glyco-diosgenin (GDN, Anatrace) at 4 ºC for 2 h. The resin was then washed by 10 column volumes of washing buffer 2 containing 25 mM HEPES, pH 7.4, 100 mM NaCl, 2 mM $MgCl_2$, 0.01% (w/v) GDN, 0.001% (w/v) CHS and 30 μM 2MeSADP. The complex protein was eluted with 5 column volumes of elution buffer containing 20 mM HEPES, pH 7.4, 100 mM NaCl, 2 mM $MgCl_2$, 0.01% (w/v) GDN, 0.001% (w/v) CHS, 50 μM 2MeSADP and 50 mM biotin. The purified complex was concentrated to 1.5 ml, and then incubate with scFv16 at a molar ratio of 1:1.5 overnight at 4 ºC. The complex was further concentrated to 500 μl and purified using a Superdex 200 Increase 10/300 GL column (GE Healthcare) pre-equilibrated in a buffer containing 20 mM HEPES, pH 7.4, 100 mM NaCl, 0.01% (w/v) GDN, 0.001% (w/v) CHS and 1 μM 2MeSADP. The complex peak fractions were collected and concentrated separately to 2.0 mg ml^{-1} using a 100-kDa cut-off concentrator (Millipore).

The cells that express the $P2Y_{12}R$–G_{i2} complex were thawed and lysed in a hypotonic buffer of 20 mM HEPES, pH 7.4, 50 mM NaCl and 2 mM $MgCl_2$ supplemented with EDTA-free complete protease inhibitor cocktail tablets (Roche). The 2MeSADP–$P2Y_{12}R$–G_{i2} complex was formed in the membranes by adding 2MeSADP to a final concentration of 10 μM and 2

units of apyrase (NEB). The lysate was incubated for 1–2 h at room temperature and the membranes were collected by centrifugation at 40,000*g* for 30 min. The membranes were solubilized in a buffer containing 20 mM HEPES, pH 7.4, 50 mM NaCl, 2 mM $MgCl_2$, 0.5% (w/v) DDM, 0.1% (w/v) CHS, 10 μM 2MeSADP, 100 μM TCEP and 2 units of apyrase (NEB) at 4 ºC for 3 h. The supernatant was isolated by ultracentrifugation at 40,000*g* for 30 min and then incubated with strep resin (IBA Lifesciences) overnight at 4 ºC. The resin was washed with 10 column volumes of wash buffer 1 containing 20 mM HEPES, pH 7.4, 50 mM NaCl, 2 mM $MgCl_2$, 0.05% DDM, 0.01% CHS and 30 μM 2MeSADP. Then the detergent was exchanged by incubating the resin with wash buffer 1 supplemented with 0.5% (w/v) lauryl maltose neopentyl glycol (LMNG, Anatrace) at 4 ºC for 2 h. The resin was then washed with 10 column volumes of washing buffer 2 containing 20 mM HEPES, pH 7.4, 50 mM NaCl, 0.01% (w/v) LMNG, 0.002% (w/v) CHS and 50 μM 2MeSADP. The protein was eluted with 5 column volumes of elution buffer containing 20 mM HEPES, pH 7.4, 50 mM NaCl, 2 mM $MgCl_2$, 0.01% (w/v) LMNG, 0.002% (w/v) CHS, 50 μM 2MeSADP and 50 mM biotin. The purified complex was concentrated to 500 μl and further purified using a Superdex 200 Increase 10/300 GL column (GE Healthcare) pre-equilibrated in a buffer containing 20 mM HEPES, pH 7.4, 50 mM NaCl, 2 mM $MgCl_2$, 0.002% (w/v) LMNG and 0.0004% (w/v) CHS. The complex peak fractions were collected and concentrated separately to 1.0 mg ml^{-1} using a 100-kDa cut-off concentrator (Millipore).

Cryo-EM data acquisition and processing

For cryo-EM studies, 3 μl of 2MeSADP–$P2Y_1R$–G_{11} and 2MeSADP–$P2Y_{12}R$–G_{i2} samples were applied to glow-discharged 200 mesh gold grids (NiTi R1.2/1.3) and vitrified using the FEI Mark IV Vitrobot (ThermoFisher Scientific). Images were collected on a 300 kV Titan Krios G3 electron microscope (FEI) equipped with a Gatan K3 Summit direct electron detector

operated at a nominal magnification of 130,000×, corresponding to a pixel size of 1.045 Å. The slit width for zero loss peak was 20 eV using GIF-Quantum LS Imaging energy filter. Movie stacks were obtained with a defocus range of –1.3 to –2.3 μm, using SerialEM (Mastronarde, 2005) with a set of customized scripts enabling automated low-dose image acquisition. Each movie stack was dose-fractionated over 32 frames by 3-s exposure with the dose rate of 2.1875 electrons per Å^2 per frame.

A total of 9,856 image stacks for the 2MeSADP–P2Y$_1$R–G$_{11}$ complex and 5,155 image stacks for the 2MeSADP–P2Y$_{12}$R–G$_{i2}$ complex were collected and subjected to beam-induced motion correction using MotionCor2 (Zheng et al., 2017). Contrast transfer function parameters for each micrograph were estimated from the exposure-weighted averages of all frames by Gctf v1.06 (Zhang, 2016), and implemented in Relion3.0 (Zivanov et al., 2018). A total of 6,184,568 particles for the 2MeSADP–P2Y$_1$R–G$_{11}$ complex and 3,723,986 particles for the 2MeSADP–P2Y$_{12}$R–G$_{i2}$ complex were auto-picked, extracted and subjected to two-dimensional (2D) classification, three-dimensional (3D) classification and initial refinement using Relion3.0. For the P2Y$_1$R complex, a dataset of 659,399 particles was subjected to 3D auto-refinement and further Bayesian polishing, resulting in a final 2.9 Å density map determined by gold-standard Fourier shell correlation using the 0.143 criteria. For the P2Y$_{12}$R complex, a dataset of 858,424 particles was subjected to the final 3D refinement and Bayesian polishing, resulting in a final density map at 3.0 Å. Local resolution estimation was performed with the Bsoft package (Heymann and Belnap, 2007) using two unfiltered half maps.

Model building and refinement

Homology models of the active P2Y$_1$R and G$_{11}$ were built using SWISS-MODEL server (Waterhouse et al., 2018) with the inactive P2Y$_1$R (PDB code: 4XNW) and the G$_{11}$ fraction in the M1R–G$_{11}$ structure (PDB code: 6OIJ) as template models. Homology models of the active

$P2Y_{12}R$ and G_{i2} were built using the inactive $P2Y_{12}R$ (PDB code: 4PXZ) and the G_i fraction in the μOR–G_i structure (PDB code: 6DDE) as template models, respectively. All models were docked into the EM density maps using Chimera (Pettersen et al., 2004) followed by iterative manual adjustment in COOT (Emsley et al., 2010) and real-space refinement in Phenix softwares (Singharoy et al., 2015). The model statistics were validated using MolProbity (Davis et al., 2007). Structural figures were prepared by Chimera (Singharoy et al., 2015), ChimeraX (Pettersen et al., 2021) or PyMOL (https://pymol.org/2/). The data processing and refinement statistics are provided in Table S1.

BRET assay using TRUPATH biosensors

The G protein activation of $P2Y_1R$ and $P2Y_{12}R$ was measured using a BRET assay performed as previously described (Olsen et al., 2020). In brief, the HEK293T cells (obtained from and certified by the Cell Bank at the Chinese Academy of Sciences) were maintained and passaged in DMEM medium containing 8% FBS, 100 μg ml^{-1} penicillin and 100 μg ml^{-1} streptomycin (Gibco-Thermo Fisher) in a humidified atmosphere at 37 °C and 5% CO_2. The cells were then plated either in a 6-well plate at a cell density of 5–6 × 10^5 cells per well or in a 10-cm dish at a cell density of 5–6 × 10^6 cells per dish. After 24 h, the cells were transfected with the plasmids of the receptor, $G\alpha_q$-Rluc8 or $G\alpha_i$-Rluc8, $G\beta_3$ and $G\gamma_9$-GFP2 at a ratio of 1:1:1:1 (500 ng per plasmid for 6-well dishes; 2,500 ng per plasmid for 10-cm dishes). PEI-Max 40000 (Polysciences) was used to assess DNA delivery to the cells at a ratio of 3 μg PEI-MAX 40000 per μg of plasmid in the OptiMEM medium (Gibco-ThermoFisher). The next day, the cells were harvested by 0.25% Trypsin-EDTA (Thermo Fisher Scientific) and plated in a poly-d-lysine-coated white 96-well assay plate (Corning) at a density of 2–5 × 10^4 cells per well. One day after plating, the growth medium was carefully aspirated from the plates and replaced immediately with 60 μl of assay buffer (Hank's balanced salt solution (HBSS), 20mM HEPES,

pH 7.4 and 0.1% BSA), followed by addition of 10 μl of 50 μM freshly prepared coelenterazine 400a (Nanolight Technologies). After a 5-min equilibration period, the cells were treated with 30 μl different concentrations of 2MeSADP (0.001 pM–10 μM) for additional 10–15 min. The plates were then read in a Synergy H1 microplate reader (Biotek Technologies) with 395 nm (RLuc8-coelenterazine 400a) and 510 nm (GFP2) emission filters. The plates were read serially eight times, and measurements from the eighth read were used in all analyses. The BRET ratios were computed as the ratio of the GFP2 emission to the RLuc8 emission. Dose-response data of the BRET assay were analyzed using Prism 8 (GraphPad). Non-linear curve fit was performed using a three-parameter logistic equation [log (agonist vs response)]. All data are presented as mean ± SEM from at least three independent experiments performed in triplicate. Statistical significance was determined by Dunnett's test.

REFERENCES

Davis, I.W., Leaver-Fay, A., Chen, V.B., Block, J.N., Kapral, G.J., Wang, X., Murray, L.W., Arendall, W.B., 3rd, Snoeyink, J., Richardson, J.S., et al. (2007). MolProbity: all-atom contacts and structure validation for proteins and nucleic acids. Nucleic. Acids Res. 35, W375-383.

Draper-Joyce, C.J., Khoshouei, M., Thal, D.M., Liang, Y.L., Nguyen, A.T.N., Furness, S.G.B., Venugopal, H., Baltos, J.A., Plitzko, J.M., Danev, R., et al. (2018). Structure of the adenosine-bound human adenosine A1 receptor-Gi complex. Nature 558, 559-563.

Emsley, P., Lohkamp, B., Scott, W.G., and Cowtan, K. (2010). Features and development of Coot. Acta Crystallogr. D Biol. Crystallogr. 66, 486-501.

Heymann, J.B., and Belnap, D.M. (2007). Bsoft: image processing and molecular modeling for electron microscopy. J. Struct. Biol. 157, 3-18.

Maeda, S., Koehl, A., Matile, H., Hu, H., Hilger, D., Schertler, G.F.X., Manglik, A., Skiniotis, G., Dawson, R.J.P., and Kobilka, B.K. (2018). Development of an antibody fragment that stabilizes GPCR/G-protein complexes. Nat. Commun. 9, 3712.

Mastronarde, D.N. (2005). Automated electron microscope tomography using robust prediction of specimen movements. J. Struct. Biol. 152, 36-51.

Olsen, R.H.J., DiBerto, J.F., English, J.G., Glaudin, A.M., Krumm, B.E., Slocum, S.T., Che, T., Gavin, A.C., McCorvy, J.D., Roth, B.L., et al. (2020). TRUPATH, an open-source biosensor platform for interrogating the GPCR transducerome. Nat. Chem. Biol. 16, 841-849.

Pettersen, E.F., Goddard, T.D., Huang, C.C., Couch, G.S., Greenblatt, D.M., Meng, E.C., and Ferrin, T.E. (2004). UCSF Chimera--a visualization system for exploratory research and analysis. J. Comput. Chem. 25, 1605-1612.

Pettersen, E.F., Goddard, T.D., Huang, C.C., Meng, E.C., Couch, G.S., Croll, T.I., Morris, J.H., and Ferrin, T.E. (2021). UCSF ChimeraX: Structure visualization for researchers, educators, and developers. Protein Sci. 30, 70-82.

Singharoy, A., Venkatakrishnan, B., Liu, Y., Mayne, C.G., Lee, S., Chen, C.H., Zlotnick, A., Schulten, K., and Flood, A.H. (2015). Macromolecular Crystallography for Synthetic Abiological Molecules: Combining xMDFF and PHENIX for Structure Determination of Cyanostar Macrocycles. J. Am. Chem. Soc. 137, 8810-8818.

Waterhouse, A., Bertoni, M., Bienert, S., Studer, G., Tauriello, G., Gumienny, R., Heer, F.T., de Beer, T.A.P., Rempfer, C., Bordoli, L., et al. (2018). SWISS-MODEL: homology modelling of protein structures and complexes. Nucleic. Acids Res. 46, W296-W303.

Zhang, K. (2016). Gctf: Real-time CTF determination and correction. J Struct Biol 193, 1-12.

Zheng, S.Q., Palovcak, E., Armache, J.P., Verba, K.A., Cheng, Y., and Agard, D.A. (2017). MotionCor2: anisotropic correction of beam-induced motion for improved cryo-electron microscopy. Nat. Methods 14, 331-332.

Zivanov, J., Nakane, T., Forsberg, B.O., Kimanius, D., Hagen, W.J., Lindahl, E., and Scheres, S.H. (2018). New tools for automated high-resolution cryo-EM structure determination in RELION-3. Elife 7.

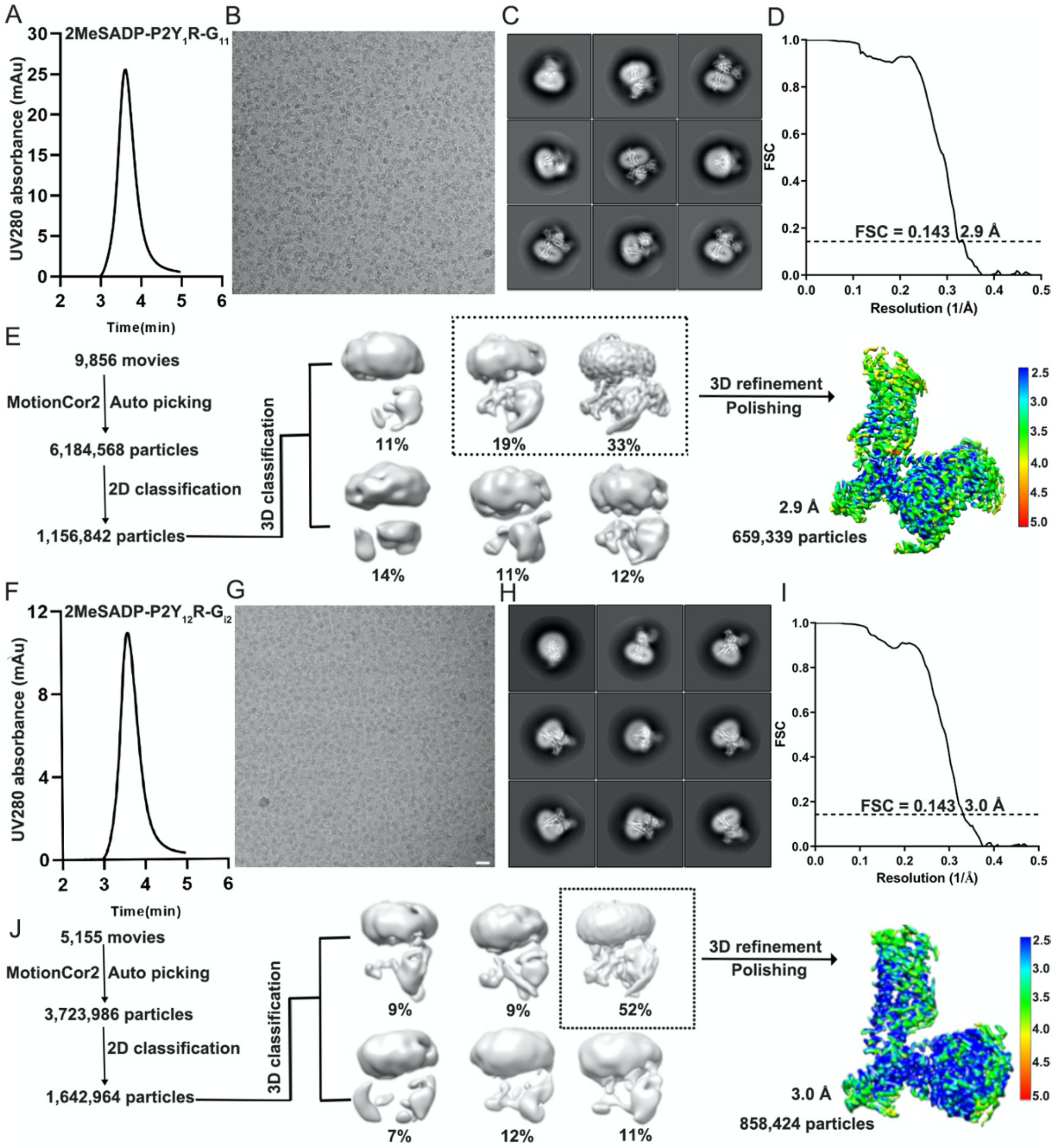

Figure S1. Sample preparation and cryo-EM data processing. (A-E) Results of the 2MeSADP–P2Y$_1$R–G$_{11}$ complex. (A) Analytical size-exclusion chromatography (aSEC) of the purified complex. (B) Representative cryo-EM image. (C) Two-dimensional (2D) averages. (D) Gold-standard Fourier shell correlation (FSC) cure showing an overall resolution at 2.9 Å. (E) Workflow of cryo-EM data processing with cryo-EM map colored according to local resolution (Å). (F-J) Results of the 2MeSADP–P2Y$_{12}$R–G$_{i2}$ complex. (F) aSEC of the purified complex. (G) Representative cryo-EM image. (H) 2D averages. (I) Gold-standard FSC curve showing an overall resolution at 3.0 Å. (J) Workflow of cryo-EM data processing with cryo-EM map colored according to local resolution (Å).

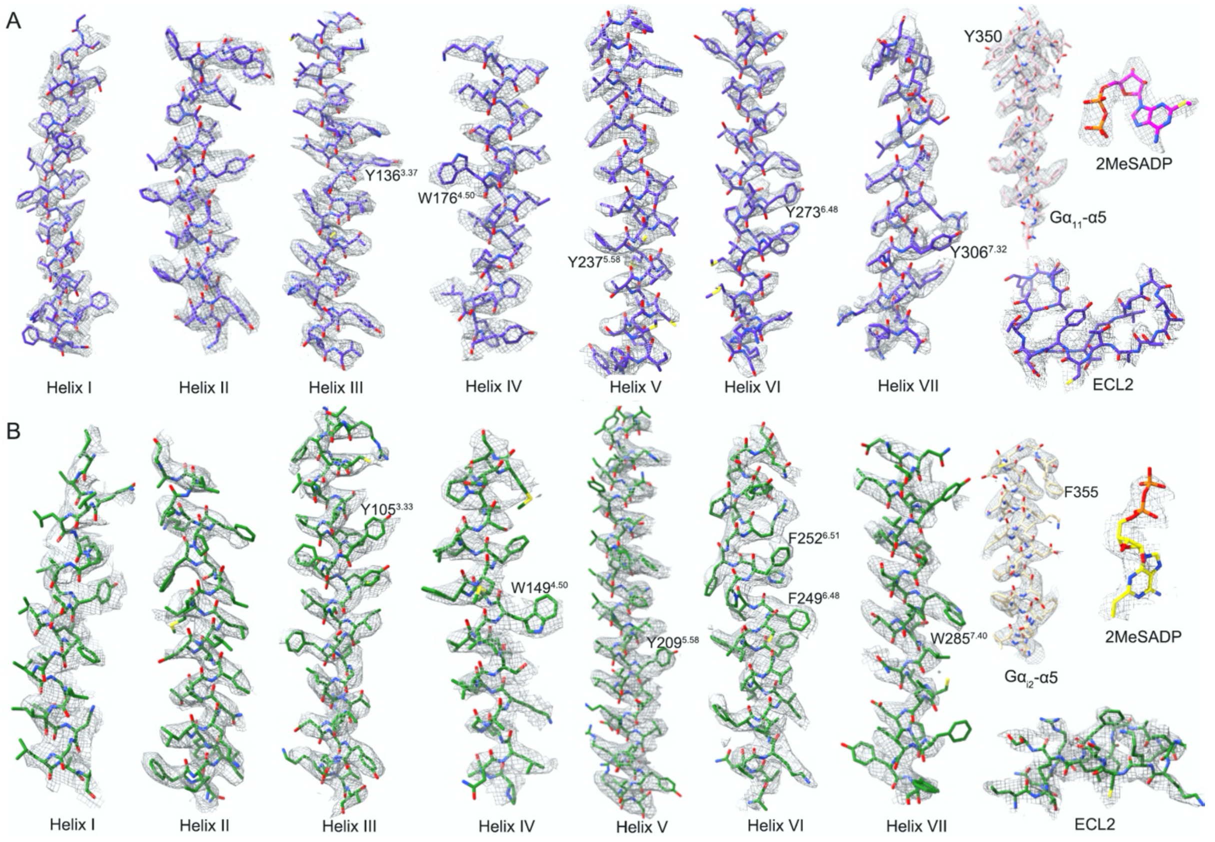

Figure S2. Electron density maps of the 2MeSADP–P2Y$_1$R–G$_{11}$ and 2MeSADP–P2Y$_{12}$R–G$_{i2}$ complexes. (A) Cryo-EM density maps and models of the 2MeSADP–P2Y$_1$R–G$_{11}$ complex are shown for the transmembrane helices, ECL2, 2MeSADP and the α5 helix of Gα$_{11}$. (B) Cryo-EM density maps and models of the 2MeSADP–P2Y$_{12}$R–G$_{i2}$ complex are shown for the transmembrane helices, ECL2, 2MeSADP and the α5 helix of Gα$_{i2}$. The models are shown as sticks and colored blue (P2Y$_1$R), green (P2Y$_{12}$R), pink (Gα$_{11}$), light yellow (Gα$_{i2}$), magenta and yellow (2MeSADP).

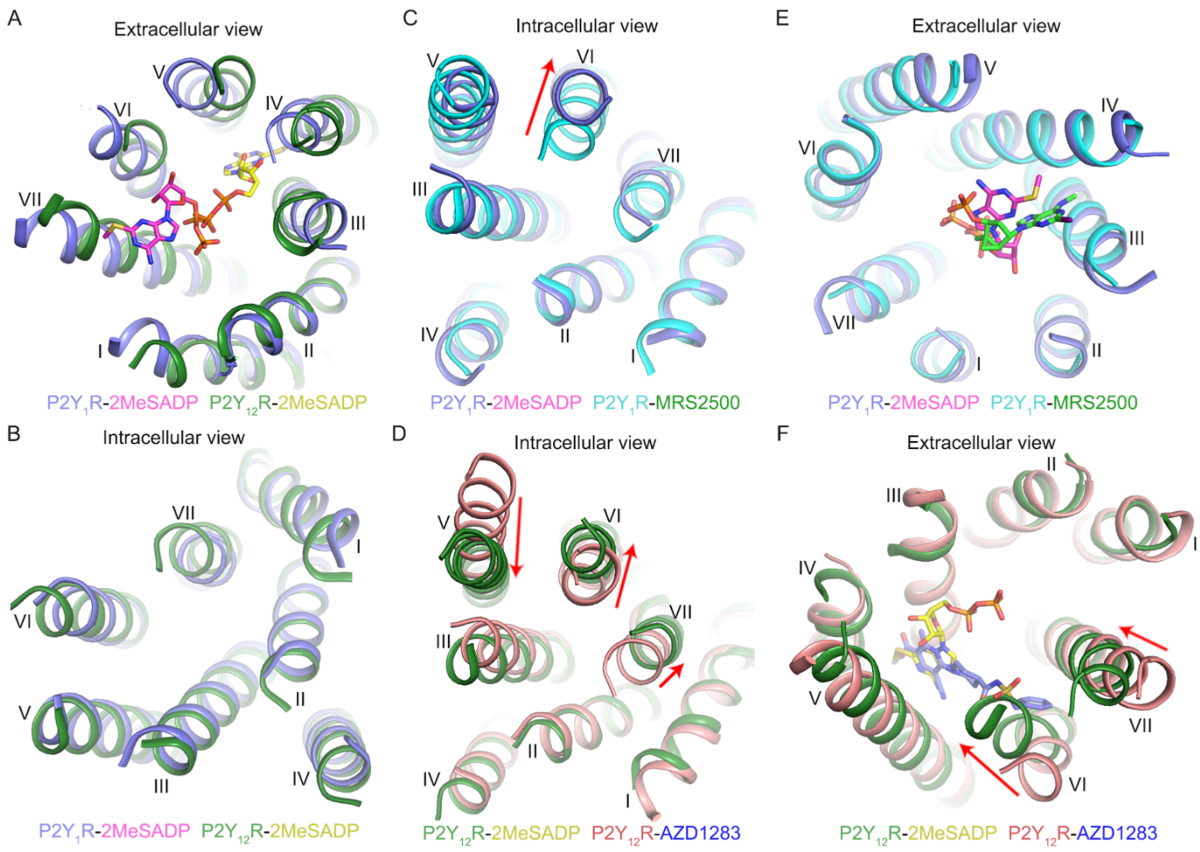

Figure S3. Comparison of the $P2Y_1R$ and $P2Y_{12}R$ structures. (A and B) Structural comparison of the transmembrane helical bundles in the 2MeSADP–$P2Y_1R$–G_{11} and 2MeSADP–$P2Y_{12}R$–G_{i2} complexes. A, Extracellular view; B, intracellular view. (C and E) Structural comparison of the transmembrane helical bundles in the 2MeSADP–$P2Y_1R$–G_{11} and $P2Y_1R$–MRS2500 complexes. C, Intracellular view; E, extracellular view. The red arrow (in C) indicates the movement of helix VI in the 2MeSADP–$P2Y_1R$–G_{11} structure relative to the $P2Y_1R$–MRS2500 structure (PDB ID: 4XNW). (D and F) Structural comparison of the transmembrane helical bundles in the 2MeSADP–$P2Y_{12}R$–G_{i2} and $P2Y_{12}R$–AZD1283 complexes. D, Intracellular view; F, extracellular view. The red arrows indicate the movements of the extracellular tips of helices VI and VII (in F) and the intracellular tips of helices V, VI and VII (in D) in the 2MeSADP–$P2Y_{12}R$–G_{i2} structure relative to the $P2Y_{12}R$–AZD1283 structure (PDB ID: 4NTJ).

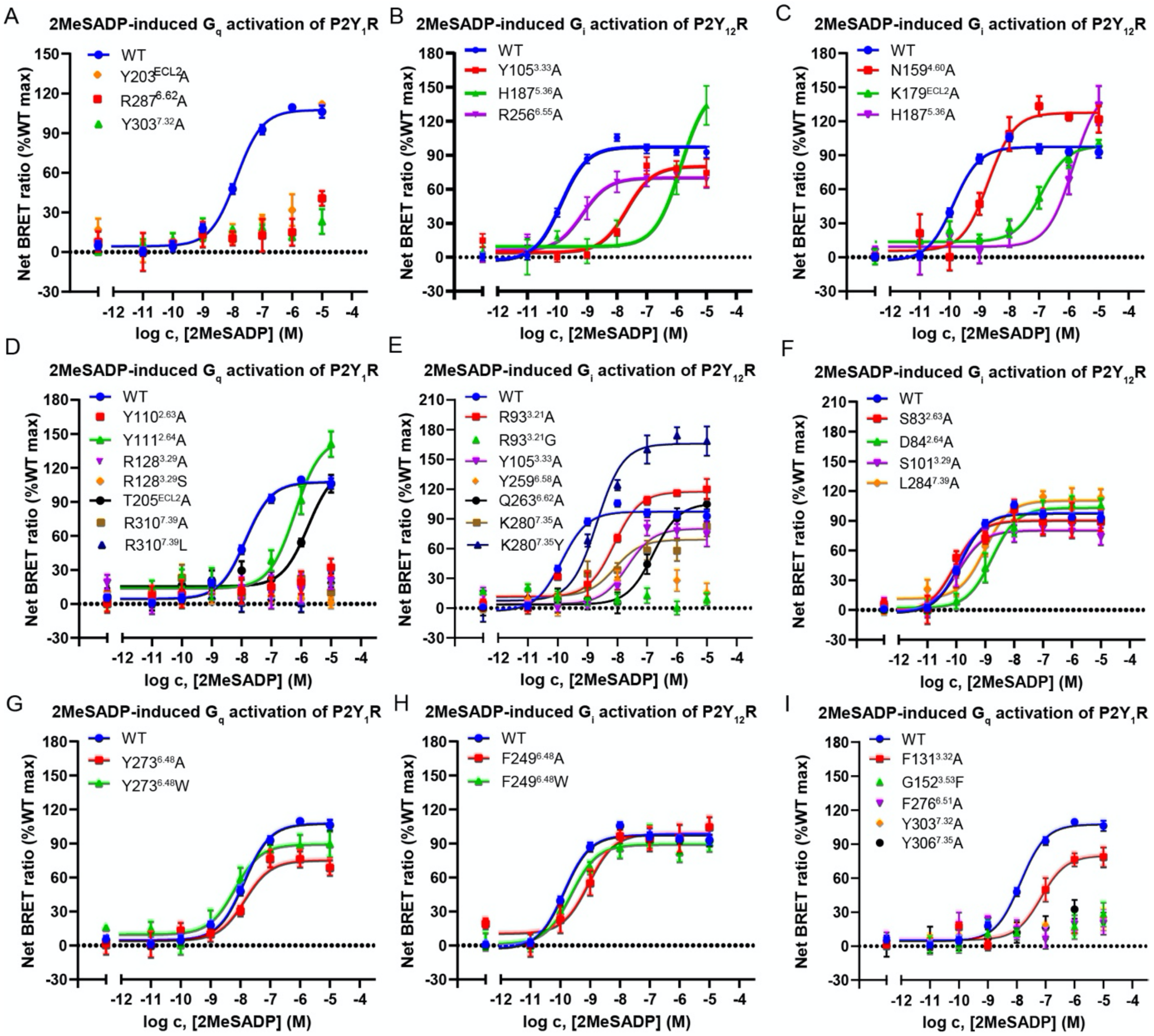

Figure S4. 2MeSADP-induced G protein activation assays of $P2Y_1R$ and $P2Y_{12}R$. (A, D, G, and I) 2MeSADP-induced G_q activation of wild-type $P2Y_1R$ (WT) and mutants. (B, C, E, F, and H) 2MeSADP-induced G_i activation of wild-type $P2Y_{12}R$ (WT) and mutants. Data are shown as mean ± SEM from at least three independent experiments performed in triplicate. Table S2 provides detailed numbers of independent experiments (*n*), statistical evaluation and expression levels.

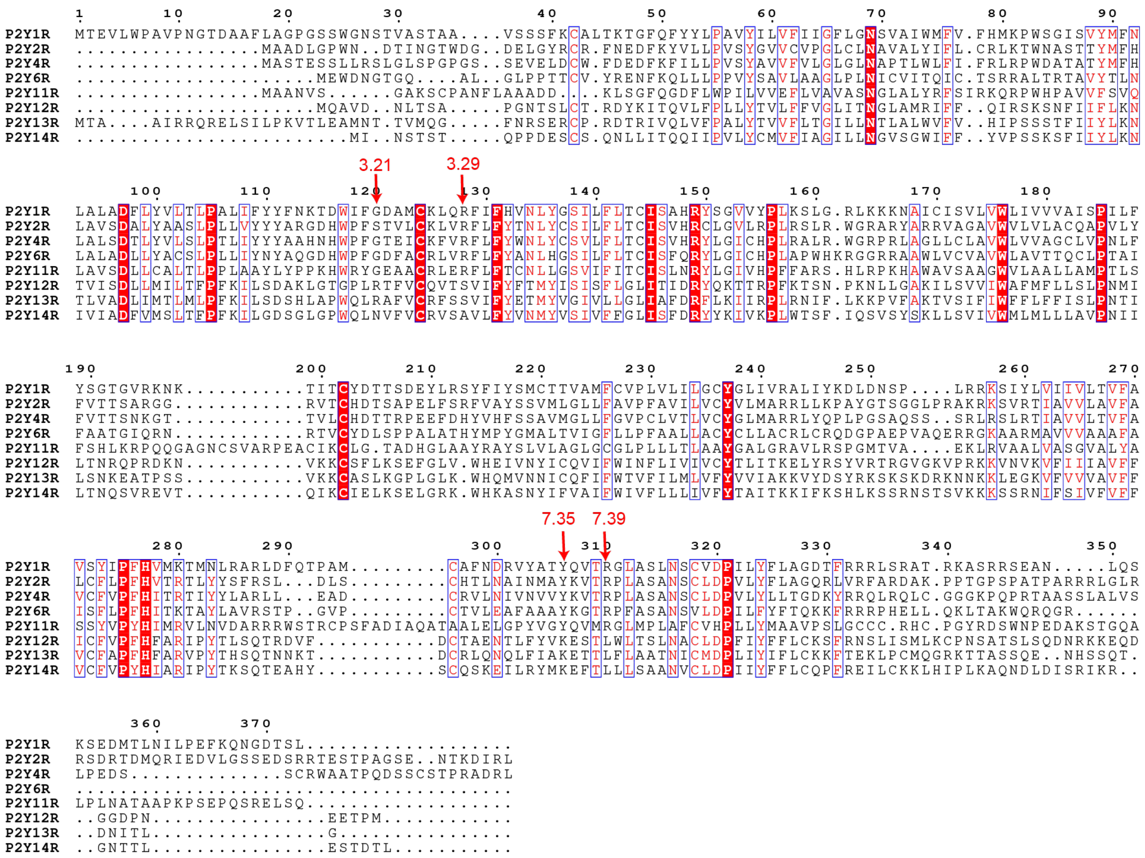

Figure S5. Sequence alignment of the human P2YRs. P2Y$_1$R-like receptors, P2Y$_1$R, P2Y$_2$R, P2Y$_4$R, P2Y$_6$R, and P2Y$_{11}$R; P2Y$_{12}$R-like receptors, P2Y$_{12}$R, P2Y$_{13}$R, and P2Y$_{14}$R. The alignment was generated using UniProt (http://www.uniprot.org/align/) and the graphic was prepared on the ESPript 3.0 server (http://espript.ibcp.fr/ESPript/cgi-bin/ESPript.cgi). Colors represent the similarity of residues: red background, identical; red text, strongly similar. The red arrows highlight the amino acids at positions 3.21, 3.29, 7.35 and 7.39.

Table S1. Cryo-EM data collection, refinement and validation statistics

	2MeSADP–P2Y_1R–G_{11} (EMDB-33503) (PDB 7XXH)	2MeSADP–P2Y_{12}R–G_{i2} (EMDB-33504) (PDB 7XXI)
**Data collection and processing**		
Magnification	130,000	130,000
Voltage (kV)	300	300
Electron exposure (e^-/Å^2)	60	60
Defocus range (μm)	–1.3 ~ –2.3	–1.3 ~ –2.3
Pixel size (Å)	1.045	1.045
Symmetry imposed	C1	C1
Initial particle images (no.)	6,184,568	3,723,986
Final particle images (no.)	659,399	858,424
Map resolution (Å)	2.9	3.0
FSC threshold	0.143	0.143
Map resolution range (Å)	2.5 - 5.0	2.5 - 5.0
**Refinement**		
Initial model used (PDB code)	4NXW, 6OIJ	4PXZ, 6DDE
Model resolution (Å)	3.2	3.2
FSC threshold	0.5	0.5
Map sharpening *B* factor (Å^2)	–88	–83
Model composition		
Non-hydrogen atoms	8,856	7,004
Protein residues	1,159	907
Ligands	1	1
B factors (Å^2)		
Protein	43.86	56.97
Ligand	72.45	71.56
R.m.s. deviations		
Bond lengths (Å)	0.002	0.002
Bond angles (°)	0.485	0.519
Validation		
MolProbity score	1.49	1.65
Clashscore	8.69	9.11
Poor rotamers (%)	0.00	0.00
Ramachandran plot		
Favored (%)	97.90	97.09
Allowed (%)	2.10	2.91
Disallowed (%)	0.00	0.00

Table S2. 2MeSADP-induced G protein activation of wild-type (WT) P2Y$_1$R and P2Y$_{12}$R and mutants using BRET assays

2MeSADP-induced G_q activation of P2Y$_1$R						
Mutants	EC$_{50}$ (nM)	EC$_{50}$ Ratio[a]	pEC$_{50}$ ± SEM[b]	E_{max}[b,c] (% of WT)	n[d]	Expression[e] (% of WT)
Wild type	13	1	7.87 ± 0.06	100 ± 3	30	100
Y110$^{2.63}$A[f]	nd	nd	nd	nd	6	65 ± 1**
Y111$^{2.64}$A	616	46	6.21 ± 0.18***	133 ± 14*	3	59 ± 1***
R128$^{3.29}$A	nd	nd	nd	nd	5	90 ± 3
R128$^{3.29}$S	nd	nd	nd	nd	3	57 ± 5***
F131$^{3.32}$A	69	5	7.16 ± 0.20*	74 ± 7	3	95 ± 2
G152$^{3.53}$F	nd	nd	nd	nd	3	80 ± 4
Y203^{ECL2}A	nd	nd	nd	nd	4	134 ± 1**
T205^{ECL2}A	1,709	131	5.77 ± 0.18***	106 ± 12	6	64 ± 2**
T206^{ECL2}A	nd	nd	nd	nd	4	24 ± 11***
Y273$^{6.48}$A	14	1	7.86 ± 0.20	71 ± 6	3	126 ± 5*
Y273$^{6.48}$W	6.5	0.5	8.19 ± 0.20	80 ± 7	4	177 ± 9***
F276$^{6.51}$A	nd	nd	nd	nd	4	11 ± 15***
R287$^{6.62}$A	nd	nd	nd	nd	4	81 ± 9
Q291^{ECL3}A	4.3	0.3	8.37 ± 0.20	112 ± 9	3	127 ± 6*
Y303$^{7.32}$A	nd	nd	nd	nd	5	60 ± 4***
Y306$^{7.35}$A	nd	nd	nd	nd	6	54 ± 6***
R310$^{7.39}$A	nd	nd	nd	nd	6	33 ± 1***
R310$^{7.39}$L	nd	nd	nd	nd	4	96 ± 1
**2MeSADP-induced G_i activation of P2Y$_{12}$R**						
Mutants	EC$_{50}$ (nM)	EC$_{50}$ Ratio[a]	pEC$_{50}$ ± SEM[b]	E_{max}[b,c] (% of WT)	n[d]	Expression[e] (% of WT)
Wild type	0.13	1	9.87 ± 0.08	100 ± 4	40	100
S83$^{2.63}$A[f]	0.087	0.7	10.00 ± 0.23	92 ± 10	6	37 ± 4***
D84$^{2.64}$A	1.7	13	8.40 ± 0.16***	101 ± 7	7	18 ± 2***
R93$^{3.21}$A	7.8	60	8.10 ± 0.14***	106 ± 6**	3	60 ± 2***
R93$^{3.21}$G	nd	nd	nd	nd	6	70 ± 1***
S101$^{3.29}$A	0.12	1	9.92 ± 0.20	80 ± 7	8	52 ± 4***
Y105$^{3.33}$A	22	186	7.65 ± 0.19***	76 ± 6	4	81 ± 4*
F106$^{3.34}$A	0.04	0.2	10.44 ± 0.30	63 ± 9*	5	80 ± 8*
N159$^{4.60}$A	2.0	15	8.70 ± 0.17***	121 ± 8	4	29 ± 5***
K179^{ECL2}A	117	898	6.90 ± 0.18***	85 ± 7	5	71 ± 3***
H187$^{5.36}$A	nd	nd	nd	nd	5	145 ± 6***
N191$^{5.40}$A	0.11	1	9.93 ± 0.17	80 ± 6	4	78 ± 7*
F249$^{6.48}$A	0.90	7	9.04 ± 0.20**	88 ± 8	7	78 ± 2*
F249$^{6.48}$W	0.23	2	9.60 ± 0.18	88 ± 7	5	55 ± 2***
R256$^{6.55}$A	0.70	5	9.17 ± 0.20	63 ± 6*	4	35 ± 3***
Y259$^{6.58}$A	nd	nd	nd	nd	5	127 ± 2**
Q263$^{6.62}$A	159	1,123	6.80 ± 0.19***	102 ± 10	5	85 ± 6
K280$^{7.35}$A	8.2	63	8.10 ± 0.31***	56 ± 8***	5	102 ± 9
K280$^{7.35}$Y	2.1	16	8.68 ± 0.13**	158 ± 10***	3	42 ± 3***
L284$^{7.39}$A	0.96	7	9.02 ± 0.17***	99 ± 7	7	33 ± 1***

[a]The EC$_{50}$ ratio, EC$_{50}$(mutant)/EC$_{50}$(WT), represents the shift between the wild-type (WT) and mutant curves, and characterizes the effect of the mutations on receptor activation.

[b]Data are shown as mean ± SEM from at least three independent experiments performed in triplicate. *$P < 0.05$, **$P < 0.01$, ***$P < 0.001$ (one-way ANOVA followed by Dunnett's post-test, compared with the response of wild type).

[c]The maximal response is reported as a percentage of the maximum effect at the wild type. nd (not determined) refers to data where a robust concentration response curve could not be established within the concentration range tested.
[d]Sample size, the number of independent experiments performed in triplicate.
[e]Protein expression levels of $P2Y_1R$ and $P2Y_{12}R$ constructs at the cell surface were determined in parallel by flow cytometry with an anti-Flag antibody (Sigma-Aldrich) and reported as per cent compared to the wild type from three independent measurements performed in duplicate.
[f]All mutations were introduced in the wild type.
